# Regulatory T Cells in HIV Infection: Can Immunotherapy Regulate the Regulator?

**DOI:** 10.1155/2012/908314

**Published:** 2012-10-15

**Authors:** Mohammad-Ali Jenabian, Petronela Ancuta, Norbert Gilmore, Jean-Pierre Routy

**Affiliations:** ^1^Chronic Viral Illnesses Service, McGill University Health Centre, 3650 St. Urbain Street, Montreal, QC, Canada H2X 2P4; ^2^Research Institute, McGill University Health Centre, Montreal, QC, Canada H3H 2R9; ^3^Department of Microbiology and Immunology, Faculty of Medicine, University of Montreal, Montreal, QC, Canada H3T 1J4; ^4^CHUM Research Center, Saint-Luc Hospital, Montreal, QC, Canada H2X 1P1

## Abstract

Regulatory T cells (Tregs) have a dominant role in self-tolerance and control of autoimmune diseases. These cells also play a pivotal role in chronic viral infections and cancer by limiting immune activation and specific immune response. The role of Tregs in HIV pathogenesis remains poorly understood as their function, changes according to the phases of infection. Tregs can suppress anti-HIV specific responses and conversely can have a beneficial role by reducing the deleterious impact of immune activation. We review the frequency, function and homing potential of Tregs in the blood and lymphoid tissues as well as their interaction with dendritic cells in the context of HIV infection. We also examine the new insights generated by recombinant IL-2 and IL-7 clinical trials in HIV-infected adults, including the immunomodulatory effects of Tregs. Based on their detrimental role in limiting anti-HIV responses, we propose Tregs as potential targets for immunotherapeutic strategies aimed at decreasing Tregs frequency and/or immunosuppressive function. However, such approaches require a better understanding of the time upon infection when interfering with Treg function may not cause a deleterious state of hyperimmune activation.

## 1. Introduction

Like Hercules, regulatory T-cells (Tregs) have the daunting task of maintaining immune tolerance while preventing inflammatory diseases. Among CD4 T cell populations, Tregs were described as playing a pivotal role in controlling autoimmune diseases and protecting host tissues from immune-mediated damage by limiting immune activation and proliferation during cancer and chronic viral infections [1–3]. Tregs play an important role in the maintenance of normal gut mucosal immunity where their inhibition has been associated with inflammatory bowel diseases [1, 4, 5]. Although the role of Tregs in HIV infection is largely unknown, recent literature is divided on whether these T-cell populations are beneficial or deleterious to patients. However, the recent progress in identifying new Tregs-specific surface markers, the discovery of the relative heterogeneity of Tregs due to the different maturation phases during their ontogeny, their fate, and the growing impact of homing receptor expression on their ability to traffic have contributed to a better understanding of the role of Tregs on immune regulation during HIV infection. In fact, depending on the phase of infection, Tregs frequency and tissue distribution change; therefore, their impact on HIV pathogenesis may vary accordingly. The recent use of the recombinant cytokines IL-2 and IL-7 in clinical trials provided evidence of the role of Tregs to modulate immune responses and is paving the way for future immune therapy strategies. 

## 2. Seventeen Years later: From a Single T-Cell Subtype to a Complex Family of Regulatory Cells

### 2.1. Tregs Phenotypes and Tissue Localization

Initially described in mice in 1995 [6], Tregs were identified in humans as cells constitutively expressing high levels of CD25 (the alpha chain of the IL-2 receptor) and the transcription factor forkhead box P3 (FoxP3) [6–8]. Results published by Seddiki et al. and Liu et al. in 2006 demonstrated that, in addition to a CD25^high^FoxP3^high^ phenotype, Tregs can be further identified by their low expression of CD127 (the alpha chain of the IL-7 receptor) [9, 10]. Therefore, the optimal way to identify the global Treg population is to describe these cells as CD3+CD4+CD25^high^CD127^low^FoxP3^high^. In 2009, Miyara et al. provided evidence that human Tregs are more heterogeneous than initially thought and could be subdivided into phenotypically and functionally distinct subpopulations [11]. Indeed, in humans, resting Tregs (CD45RA^+^FoxP3^low^) and activated Tregs (CD45RA^−^FoxP3^high^) showed *in vitro* suppressive properties, while Treg memory cells (CD45RA^−^FoxP3^low^) exhibited nonsuppressive function and low secretion of cytokines [11]. 

The FoxP3+CD25^high^ natural Tregs derive directly from the thymus [12]. These cells can be identified by their expression of the adhesion molecule CD31 and can differentiate into natural Treg effectors upon activation in the periphery [13]. However, induced (or adaptive) Tregs are differentiated from native CD4 T cells in inflammatory tissues (outside the thymus) [12] and their phenotype and their frequency vary under different pathological conditions. Induced Tregs include interleukin-10- (IL-10-) producing Tregs (Tr1), transforming-growth-factor (TGF-*β*-) expressing Tregs (Th3 cells) [14, 15], and CD39^+^ effector/memory Tregs [16]. Retinoic acid, a metabolite of vitamin A, acts as a key regulator of TGF-*β*-dependent immune responses; it inhibits the IL-6-driven induction of proinflammatory T helper (Th) 17 cells and promotes differentiation of anti-inflammatory Tregs [17]. In the intestinal environment, dendritic cells (DC) from mesenteric lymph nodes and the small intestine play an important role in the peripheral conversion of Tregs because they are a source of TGF-*β* and retinoic acid [18]. DC in draining lymph nodes are also able to locally activate Tregs [19]. Homing of Tregs into lymphoid versus non-lymphoid compartments may influence their immunosuppressive functions and homeostatic properties. Indeed, it has been suggested that Tregs impact immune responses via selective migration and homing at sites where regulation is required such as tumor sites, transplanted organs, and inflammatory tissues [20]. However, further studies are needed to characterize tissue-specific Tregs subsets with as particular focus on mucosal Tregs in humans that may play distinct functions under constitutive and pathological conditions. 

Natural Tregs mature in the thymus and the majority of these cells rapidly shift from a naïve phenotype (CD45RA+CD62L^+^CCR7^+^CD31^+^) to a memory phenotype (CD45RO^+^) when released into the periphery [7]. Tregs possess a preferential ability for homing into lymphoid organs and sites of inflammation, where they were initially identified. Thereafter, Tregs were identified in nonlymphoid tissues even in the absence of any overt inflammation [21]. To function successfully, Tregs must be able to modulate the activities of a wide variety of cellular components of both innate and adaptive immune responses. This depends on their ability to come into physical proximity with their targets by migrating into specific tissues [3]. The chemokine receptors CCR7, CCR5, CCR6, and CCR9 play key roles in regulating Tregs localization into lymph nodes, inflamed tissues, Payer's patches, and the small intestine, respectively, while the adhesion molecules CD31/PECAM-1 and integrin *α*4*β*7 facilitate Tregs recruitment across the thymic and intestinal vascular endothelia [22, 23].

### 2.2. Tregs-Mediated Immune Suppression

Recent studies suggest that, upon activation, Tregs showed functional plasticity to acquire a helper/effector memory phenotype and to home to inflammatory sites [24, 25]. Despite their critical role in maintaining peripheral tolerance, Tregs isolated from noninflamed lymphoid tissues or blood of healthy individuals are functionally quiescent or resting and must be activated to develop functional suppressive activity *in vitro* [26]. This obligatory requirement for activation implies that tissue microenvironmental cues must induce resting Tregs to acquire and maintain suppressive activity. The *in vitro *studies showed that only Tregs activated through TCR have a suppressive function [27]. 

**Figure 1:**
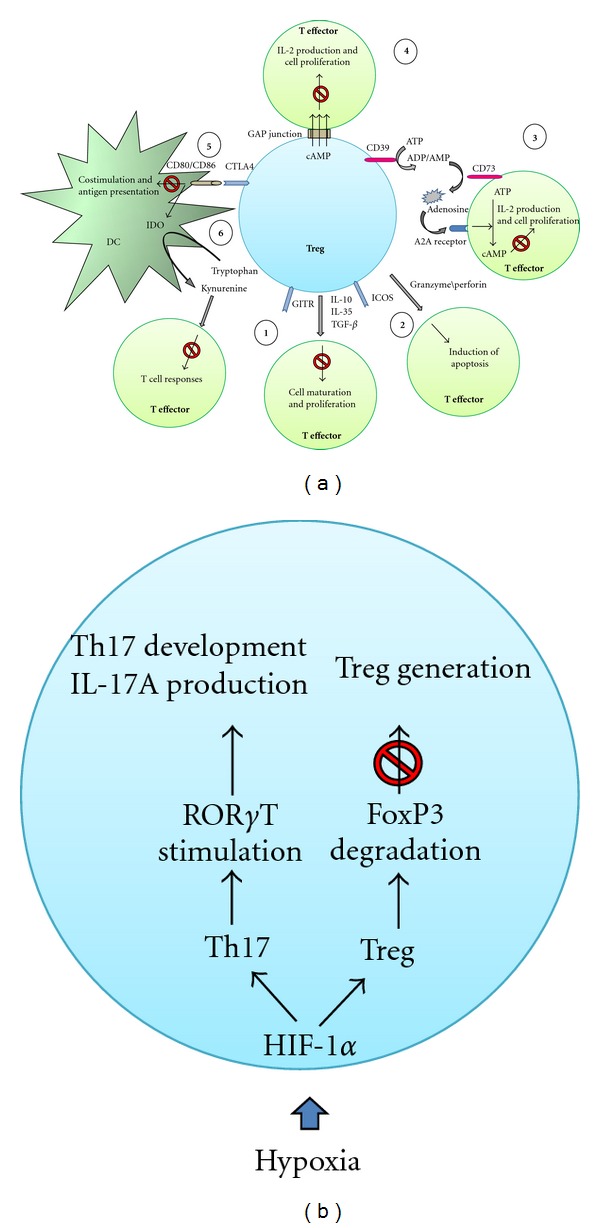
The Treg regulatory pathways. (a) The ability of Tregs to induce immune suppression is mediated via (1) inhibitory cytokines; (2) induction of apoptosis by granzyme/perforin pathway; (3) CD39/CD73/adenosine pathway; (4) direct transfer of cAMP via GAP junction; (5) inhibition of DC function by CD80/CD86 and CTLA-4 interaction; and (6) the catabolism of tryptophan via IDO enzyme. (b) Dual function of HIF-1*α* in regulation of Treg and Th17 cells.

Tregs use various mechanisms for their immunomodulatory functions (Figure 1(a)). *In vitro* studies have shown that activated Tregs suppress immune responses through the secretion of immunosuppressive cytokines such as IL-10, transforming growth factor-*β* (TGF-*β*) and IL-35 [14, 15, 28]. The expression of inducible costimulator (ICOS), as a CD28 homolog, is strongly correlated with IL-10 production [29]. ICOS also controls the pool size of effector/memory T cells and Tregs in the steady state as well as in Ag-specific immune reactions by regulating the survival of T cells [30]. Glucocorticoid-induced TNFR-related proteins (GITR), members of the TNF receptor, play an important role in regulating the immunosuppressive function of Tregs [31, 32]. Tregs contribute to the overall size and quality of the humoral response by controlling homeostasis of the germinal center B-cell pool via GITR and IL-10 [33].

Functional studies using light microscope imaging have shown that antigen recognition leads to the formation of immunological synapses at the interface between the DC, Tregs, and other T cells [34]. In the absence of proinflammatory stimuli, Tregs and naive CD4 T cells interact differently with DC. Neuropilin-1, which is expressed by most Tregs but not by naive CD4 T cells, promotes prolonged interactions with immature DC, resulting in higher sensitivity to limiting amounts of antigen [35]. Natural Tregs can downregulate the CD80 and CD86 expression on DC and control antigen-presenting cell function via cytotoxic T lymphocyte antigen 4 (CTLA-4) [36]. Of interest, CTLA-4 blockade decreases expression of the tryptophan- (Trp-) depleting enzyme indoleamine-2,3-dioxygenase (IDO) and the level of the suppressive cytokine TGF-*β* in tissues [37]. Several studies demonstrated that Tregs play an important role in tumor clearance and in controlling viral inflammation by inducing the apoptosis of target cells via granzyme B/perforin pathway [38, 39]. 

Tregs may also play their inhibitory role using ecto-enzymes including the ectonucleoside triphosphate diphosphorylase-1 (CD39) and the ecto-5′-nucleotidase (CD73) for their suppressive activity. These enzymes hydrolyse extracellular pools of inflammatory ATP into adenosine diphosphate (ADP) and/or adenosine monophosphate (AMP) to adenosine [27]. Extracellular adenosine is known to be an important physiological regulator of the immune response [40, 41] by inhibiting T-cell proliferation and IFN-*γ*/IL-2 production [42]. These effects are mediated through the adenosine-receptor A2A (A2AR) by stimulating the generation of intracellular cyclic AMP (cAMP) [42]. Glucocorticoid-induced TNFR-related proteins (GITR), members of the TNF receptor, play an important role in regulating the immunosuppressive function of Tregs [31, 32]. Tregs contribute to the overall size and quality of the humoral response by controlling homeostasis of germinal center B cell pool via GITR and IL-10 [33].

FoxP3 is the master regulator of Tregs function through interactions with a variety of transcription factors including the nuclear factor of activated T-cells (NFAT), histone deacetyltransferase (HDAC), and the nuclear factor kappa B (NF-*κ*B) [43–45]. FoxP3 also inhibits the transcription factor ROR*γ*t to influence the balance between Tregs and interleukin- (IL-)-17-producing effector T cells, known as Th17 cells [46]. Diverse mechanisms are used by Tregs to mediate suppressive activities. During inflammation, the insulted tissue often responds to low level of oxygen (hypoxia) by inducing a transcription factor called hypoxia-inducible factor-1alpha (HIF-1a) [47] (Figure 1(b)). This transcription factor is known to stimulate metabolism under anaerobic condition and recent reports provided evidence for its involvement in regulating Tregs homeostasis [47] and controlling the Th17/Tregs balance in favor of Th17 proliferation via ROR*γ*t activation [48, 49]. Both inflammation and hypoxia create a complex microenvironment that can induce and bias Tregs numbers and function. 

## 3. Tregs in HIV Infection: Friends or Foes?

### 3.1. Tregs and HIV Disease Progression

The progressive loss of CD4 T-cells together with the dysfunction of antigen-specific T cell responses in the context of chronic immune activation represent hallmarks of HIV infection [50, 51]. It has been demonstrated that non-Tregs CD4 T cell subsets play different functions during viral infections, notably in HIV infection, and their absolute numbers were inversely correlated with disease progression [52]. It has been shown that in SIV infection the expression of Tregs in the ileal lamina propria was significantly decreased at all stages of infection [53]. In contrast, Treg in SIV-infected and uninfected macaques was comparable in lymph nodes and peripheral blood [53]. A recent study on SHIV89.6-immunized rhesus macaques has shown a transient increase in the frequency of circulating Treg. Indeed, three days after SIV vaginal challenge, SHIV-immunized monkeys had significantly more Tregs in the vagina than the unimmunized animals. But in contrast, all the SIV infected animals had Treg depletion in different tissues 14 days after SIV infection associated with an increase of their suppressive function [54].

The deleterious or beneficial effects of Tregs in HIV pathogenesis remain controversial as these cells show divergent functions. The imbalance between effector T cells and Tregs can create a predominantly Tregs compartment, which hampers efficient effector T-cell responses, that have been observed in both cancer and chronic viral infections [55]. HIV infection induces an expansion of the relative frequency peripheral Tregs but not their absolute number due to the depletion of CD4 T cells, in acute and chronic phases of the infection [56–60]. Tregs frequency is correlated with cellular immune activation and CD4 T-cell lymphopenia [59–61]. Tregs frequency is also increased in the gut mucosae of HIV-infected patients with high viral loads [61]. The expansion of Tregs in mucosal tissues, together with the maintenance of their suppressive ability during chronic HIV infection, potentially contributes to viral persistence [61]. Tregs can suppress HIV-specific CD4 and CD8 T-cell immune responses by inhibiting cytokine production and cell proliferation *in vitro* [62, 63]. The *in vivo* augmentation of Tregs frequency is accompanied by a diminution of HIV-specific T-cell responses [64]. It has been reported recently that CD39+ Tregs are expanded in HIV infection and these cells are involved in HIV pathogenesis and AIDS progression by inhibiting HIV-specific responses [65, 66]. Indeed, the effector T cells from untreated HIV-infected patients are more sensitive to adenosine generated by CD39, as compared to HIV negative controls, due to a higher A2AR expression, the receptor of adenosine [65, 67]. The expansion of the CD39+ Tregs subset correlates positively with the level of immune activation and negatively with CD4 T-cell counts in HIV-infected subjects [65, 66]. CD39+ Tregs also inhibit IL-2 production through a CD39/adenosine/cAMP pathway via epigenetic regulation of IL-2 gene expression [67]. A recent study showed that naïve Tregs numbers were essentially preserved, whereas effector Tregs were consistently affected during HIV infection. Of particular interest, the effector but not total or naïve Tregs numbers negatively correlated with the magnitude of HIV-specific CD8 T-cell responses [64] suggesting a deleterious role of Tregs in HIV-pathogenesis by diminishing HIV immunity.

 On the other hand, Tregs can protect individuals from the deleterious effects of HIV-induced chronic immune activation [68, 69]. Tregs may inhibit HIV replication in conventional T cells during the early stages of HIV infection through a direct transfer of cAMP to conventional T cells via gap junctions [70]. In HIV-exposed uninfected people, low levels of immune activation are associated with an increase in Treg frequency suggesting that Tregs may contribute to HIV resistance by controlling levels of T cell activation and consequently by minimizing the pool of cells that are susceptible to infection [71]. Recent studies found that the frequency of Tregs in HIV elite controllers and long-term nonprogressors is similar to that in uninfected healthy subjects [66, 72, 73]. Low frequencies of Tregs in HIV elite controllers may contribute to an effective adaptive immune response, but it may also contribute to a poor control of immune activation that can lead to progressive CD4 depletion [74]. Despite quantitative changes in Tregs, HIV infection is not associated with impairment of *ex vivo* suppressive function of Tregs from HIV controllers and from untreated chronically infected patients [75]. More recently, it was shown that HIV-specific CD8 T cells from elite controllers restricted by protective HLA-B*27 and HLA-B*57 alleles are resistant to Tregs and can evade Tregs-mediated suppression by directly killing neighboring Tregs in a granzyme B-dependent manner [76]. 

### 3.2. Th17/Treg Imbalance and Mucosal Immunity in HIV Infection

Gut-associated lymphoid tissues (GALT) are major sites of HIV replication and CD4+ T cell depletion. Among CD4+ T cell subsets, Th17 cells play a critical role in maintaining mucosal immunity against pathogens [77]. Tregs and Th17 cells differentiate from common precursors when naive. Indeed, TGF-*β* induces the differentiation of Tregs, whereas TGF-*β* in combination with IL-6 or IL-21 leads to the differentiation of Th17 cells [17, 78]. HIV infection is associated with a rapid depletion of Th17 cells [79]. The progressive loss of Th17 cells results in a breakdown of mucosal immunity and an increase in microbial translocation across the gastrointestinal mucosa fuelling local and systemic immune activation.Tregs may aggravate this effect by suppressing virus-specific immune responses, thereby contributing to the loss of Th17 cells in mucosal tissue, which in turn will increase immune activation induced by microbial translocation in the gut [80]. The Th17/Treg balance has a major impact on gut mucosal immunity preventing the microbial translocation from the lumen to the lamina propria [81]. 

As emphasized earlier, IDO has a major role in the peripheral generation of Tregs under physiological or pathological conditions. Recently, a new mechanism has been identified that intrinsically links IDO activity and the differentiation of Treg versus Th17 cells from naïve T cells. Increased catabolism of Trp by IDO has a suppressive consequence on T cell responses in pregnancy, autoimmune disease, viral infections, and cancer [81]. It has been reported that the tryptophan (Trp) catabolite kynurenine, a by-product of IDO activity, can directly induce the over-expression of FoxP3 and the generation of Tregs, while suppressing the generation of Th17 cells by downregulating the expression of the Th17-specific ROR*γ*t transcription factor [82–85]. Other studies also reported that IDO influences the balance between Th17/Treg cells by upregulating the FoxP3 expression and diminishing Th17 cell frequency [86–88]. *In vitro* findings have shown that another tryptophan catabolite, 3-hydroxyanthranilic acid, can also upregulate FoxP3 expression and reduce the Treg/Th-17 balance [89]. Indeed, the pathogenic SIV infection leading to AIDS in rhesus macaques was associated with a change in the Th17/Treg balance, whereas this balance was maintained in nonpathogenic SIV infection, where progression to AIDS is not observed despite high levels of viral replication (i.e., African green monkeys) [90]. An altered Th17/Treg balance in SIV-infected rhesus macaques was directly linked to an increased IDO activity. In the nonpathogenic model of SIV infection, the IDO activity in activated DC is downregulated though IFN-*γ* signaling and Toll-like receptor (TLR) stimulation by SIV and bacterial antigens, while in pathogenic SIV infection as well as in chronic HIV infection, IFN-*γ* responses remain high, leading to the persistence of elevated IDO activity [89, 90]. Recent findings suggest an increase of IDO activity in HIV infection is associated with disease progression as well as an imbalance of Th17 cells and Tregs in both peripheral blood and in rectosigmoid mucosal tissue [81, 89, 90]. Our recent data in HIV elite controllers show that plasma levels of Trp are similar to those found in antiretroviral-therapy- (ART-) naïve chronically HIV-infected subjects, whereas the levels of Trp catabolites in HIV elite controllers are similar to those found in patients successfully treated with ART as well as in healthy uninfected subjects, suggesting a different Trp metabolism in elite controllers [91]. In line with these observations, we found that the Th17/Treg ratio is similar in HIV elite controllers and uninfected individuals [72] thus emphasizing the critical importance of the Th17/Treg balance in controlling HIV disease progression. 

### 3.3. Impact of ART on Treg Function

Recently, it has been shown in chronic Hepatitis B (HBV) that inhibition of viral replication by anti-HBV drugs is associated with diminished Treg expression [92]. However, the impact of ART on Treg frequency in HIV-infected patients remains controversial. A recent longitudinal study has shown that Treg frequencies were normalized by ART [57] and that the proportion of Tregs increased as a result of immune activation following ART interruption [93]. Other studies reported that levels of Treg frequency in ART-treated HIV patients remained significantly higher compared to those in healthy subjects [65, 66]. Long-term ART may normalize Th17 frequency as well as the Th17/Treg ratio in sigmoid colon samples when compared to that in uninfected healthy subjects [79]. However, there is evidence in the literature that Th17 cells are only partially restored by ART in the GALT [94, 95]. 

Taken together, according to the phase of infection and the level of immune activation Tregs may play a dual role in HIV infection in which there is a fragile balance between “friend” and “foe", namely, by reducing immune activation and by inhibiting HIV-specific T-cell functions, respectively. In our opinion, the conflicting roles reported in the literature for Tregs in HIV infection may be explained in part by (i) the suboptimal phenotypic markers used to identify Tregs; (ii) the absence of data on the immunosuppressive function of Tregs subsets including naïve, central, and effector memory cells; (iii) the paucity of data on migratory properties and tissue distribution of Tregs; and (iv) the lack of markers to properly assess the influence of immune activation and exhaustion associated with Treg functions.

 In addition to ART, new HIV immunotherapy investigations have shown that the frequency of Tregs may be influenced by immunotherapeutic interventions. 

## 4. Treg as a Potential Target for HIV Immunotherapy 

### 4.1. Strategies to Modulate the Interplay between Tregs and DC

Programmed death-1 (PD-1) is an important inhibitory pathway in regulation of T-cell receptor signaling, notably in chronic viral infections. It has been shown that the combination blockade of the PD-1 and CTLA-4 may reduce Treg activity in cancer [96]. In chronic HCV infection, PD-1 and programmed death ligand 1 (PDL-1) have an important role in the negative regulation of Treg numbers as well as Treg ability to suppress T-cell responses [97, 98]. PD-1 is upregulated during HIV infection, mainly on HIV-specific T cells, and PD-1 expression levels are correlated with both viral load, reduced cytokine production, and reduced proliferation capacity of HIV-specific CD8 T cells [51, 99]. The influx of microbial products and the increased production of inflammatory cytokines in HIV patients induces. The upregulation of PD-1 in monocytes, resulting in IL-10 production and reversible CD4 T cell dysfunction during HIV infection [100]. Blocking PD-1 engagement to PD-L1 enhanced the capacity of HIV-specific CD8 T cells to survive and proliferate and increase their HIV-specific responses [51]. In HIV-infected patients, the frequency of PD-1^+^ and CTLA-4^+^ T-cells is higher in the GALT when compared to the peripheral blood [101]. Amarnath et al., have shown that Tregs promote the generation of myeloid DC and Tregs-conditioned DC-induced PD-L1 expression *in vivo *on effector T cells [102]. Importantly, the PDL/PD1 axis converts human Th1 cells into regulatory T cells [103]. Furthermore, follicular Tregs which express high levels of both FoxP3 and PD-1 appear to repress follicular Th cells, inhibit B-cell selection in germinal centers, and may play a role in terminating germinal center responses [104]. Altogether, these findings suggest that Tregs may be a potential target in immunotherapeutic strategies focusing on PD-1/PDL-1 blockage.

Given the ability of IDO enzyme activity to influence the Th17/Treg balance and to enhance the suppressor activity of Tregs, modulating the activity of this enzyme is of therapeutic interest. In several types of cancer, an increased IDO activity was an independent predictor of poor clinical outcome [105] and some IDO-inhibiting drugs are currently being assessed in antitumor clinical trials [105, 106]. Results generated in an animal model of HIV-1 encephalitis demonstrated that manipulating IDO activity using the inhibitor 1-methyl-d-tryptophan (1-MT) enhances the generation of HIV-specific cytotoxic T lymphocytes leading to elimination of HIV-infected macrophages in the brain [107]. In other studies IDO appears to synergize with ART in controlling viral replication in plasma and lymph nodes of SIV-infected rhesus macaques [108]. A combination blockade of CTLA-4 and IDO transiently reduces the Kyn/Trp ratio, increases Th1 cell proliferation, and blocks Treg cell functions. However, this intervention resulted in fulminant diabetes associated with severe lymphocyte infiltration in the pancreas [109]. Further studies are required to elucidate the potential benefits of IDO inhibitors in the context of HIV immunotherapy.

### 4.2. Treg and DC-Based Immunotherapy

Development of DC-based therapeutic vaccination in SIV and HIV infection has shown some encouraging results [110]. At least 10 clinical trials are already reported in the literature and they provide evidence that DC-based immunotherapy in HIV-infected individuals can elicit HIV-specific immunological responses. Some of these studies reported beneficial virological responses to immunization [110]. Lu et al. reported that immunization of rhesus macaques with AT-2-inactivated SIVmac251-loaded autologous DC resulted in a 50-fold decrease of SIV DNA and a 1,000-fold decrease of the SIV RNA levels in the peripheral blood [111]. The same group reported that monocyte-derived DC loaded with aldrithiol-2-inactivated autologous virus-stimulated proliferative responses of autologous CD4 and CD8 T cells in HIV-infected patients. In addition, proviral DNA viral loads and HIV-1 RNA levels were significantly decreased in autologous T cells expanded with virus-pulsed DC [112]. Our team has reported a partial to complete restoration of HIV-specific proliferative immune responses in successfully ART-treated HIV patients receiving a personalized DC-based vaccine (AGS-004) produced from autologous monocyte-derived DC electroporated with RNA encoding CD40L and HIV antigens [113]. Recent investigations by our group show that Tregs frequency and immune activation markers (CD38/HLA-DR/PD-1) are not affected by this DC-based vaccine. Overall, DC-based immunotherapy appears to have no deleterious effect on expansion of immunosuppressive Tregs and nonspecific immune activation (M. A. Jenabian et al., personal data).

### 4.3. Tregs and Cytokine-Based Clinical Trials

IL-2 is produced almost exclusively by activated T cells. It facilitates Th1/Th2 differentiation and expands CD8 memory T cells, as well as NK cells, while inhibiting TGF-*β*/IL-6-dependent Th17 cell differentiation of naïve T cells [114–116]. Strong evidence suggests that IL-2 is an important stimulatory signal for Tregs development and function [117]. Tregs suppress T-cell responses by downregulating IL-2 synthesis but paradoxically also require IL-2 for acquisition of function [67, 117–119]. Mice deficient in IL-2R*α* (CD25) or IL-2R*β* (CD122) have reduced numbers of Tregs and die from inflammatory bowel diseases [120]. In humans, the genetic deficiency in CD25 induces Treg dysfunction resulting in the IPEX syndrome, characterised by immune dysregulation, polyendocrinopathy, enteropathy, and X-linked syndrome [120]. Human Tregs and effector T cells reciprocally regulate death/growth arrest by differentially utilizing the granzyme-perforin pathway depending on IL-2 concentrations [121]. IL-2 was the first cytokine to be used as an immunotherapy agent in two large clinical trials: ESPRIT and SILCAAT. Despite a substantial and sustained increase in the CD4 T cell count, IL-2 plus ART yielded no clinical benefit in either study when compared to ART alone [122]. The most plausible explanation appears to be the sustained induction of a newly expanded population of immunosuppressive Tregs following IL-2 administration [123]. In these trials, analyses of IL-2-expanded T cells showed a predominant increase of Tregs with a CD4^+^FoxP3^+^CD127^low^CD25^high^ phenotype. These IL-2-expanded cells exhibit molecular and suppressive functions characteristic of Tregs [123]. Consistently, in a recent study, clinical improvement in an autoimmune disease, known as HCV-induced vasculitis, was observed following IL-2 administration as the frequency of Tregs was increased [124]. 

**Figure 2:**
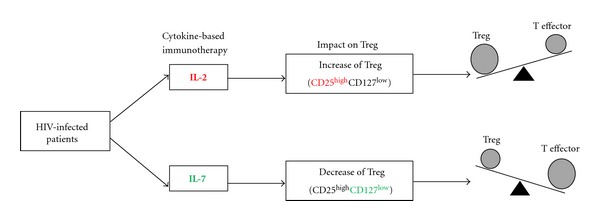
The impact of cytokine-based immunotherapy on Treg in HIV infection. The IL-2 immunotherapy results predominantly in the expansion of Treg, which express high levels of the IL-2 receptor chain CD25. In contrast, IL-7 immunotherapy may preferentially favour the effector T cell population without inducing Tregs because Tregs express low levels of the IL-7 receptor chain CD127.

Following these disappointing findings of IL-2 clinical trials, a novel IL-7 immunotherapy was developed for HIV infection. This immunotherapeutic approach was inspired by results generated in animal models showing that exogenous IL-7 administration increases CD4 T cell counts in the absence of immune activation [125–127]. IL-7 is a major regulator of T cell homeostasis produced by stromal cells in thymus and in lymphoid organs [128]. This cytokine is involved in survival, proliferation and maturation of T cells [129] and acts through the heterodimeric IL-7 receptor (IL-7R). This receptor comprises a cytokine-specific binding IL-7R alpha-chain (CD127) and a signaling common gamma-chain (CD132), the latter being also shared by the receptors for other cytokines like IL-2 and IL-15 [128]. An inverse relationship between circulating IL-7 levels and CD4 T cell counts was consistently found in various clinical settings including cancer and HIV infection [130–132], suggesting that IL-7 plays a unique role in maintaining lymphocyte homeostasis. In chronic HIV infection, high levels of IL-7 are observed predominantly when CD4 counts diminish below 200 cells/mm^3^ thus suggesting the possibility that endogenous IL-7 alone may not be sufficient to counteract T cell depletion [133]. A recent study has shown that IL-7 immunotherapy can expand gut T lymphocyte numbers, predominantly central memory CD4+ T cells, but the numbers of Th17 cells in the gut did not change significantly [134]. This finding is intriguing considering the fact that CD4 T cells expressing the Th17 marker CCR6 express the highest levels of CD127 [34]. More recently, a phase I/IIa trial of IL-7 showed a relative decrease of Tregs within the total CD4+ T-cell population in blood [135], a finding consistent with the low-to-undetectable expression of CD127 on Tregs [10]. Collectively, these results suggest that IL-7-based immunotherapy may increase CD4 T-cell counts without increasing Treg frequency (Figure 2). 

## 5. Conclusion 

The importance of Treg contribution in HIV pathogenesis is increasingly recognized. Indeed, Tregs appear to contribute to the control of viral replication during the short phase of primary infection while appearing to have a deleterious impact in the chronic phase of infection by inhibition of HIV specific immune responses. Future studies that examine new and reliable phenotypical Treg definitions, their trafficking and homing in different lymphoid tissue, and the immunomodulatory functions of Treg subsets are fundamental to further understanding of the role of Treg in HIV pathogenesis. New immunotherapeutic strategies, notably cytokine therapy, will provide insight into the circumstances whereby Treg can be a “friend” by promoting healthy outcomes in HIV infection.
